# Exploring the Role of Nanoparticles in Enhancing Mechanical Properties of Hydrogel Nanocomposites

**DOI:** 10.3390/nano8110882

**Published:** 2018-10-29

**Authors:** Josergio Zaragoza, Scott Fukuoka, Marcus Kraus, James Thomin, Prashanth Asuri

**Affiliations:** 1 Department of Bioengineering, Santa Clara University, Santa Clara, CA 95053, USA; j1zaragoza@scu.edu (J.Z.); sfukuoka@scu.edu (S.F.); mkraus1@scu.edu (M.K.); 2 Department of General Sciences, Northwest Florida State College, Niceville, FL 32578, USA; thominj@nwfsc.edu

**Keywords:** hydrogel nanocomposites, elastic modulus, rotational rheology, pseudo-crosslinking

## Abstract

Over the past few decades, research studies have established that the mechanical properties of hydrogels can be largely impacted by the addition of nanoparticles. However, the exact mechanisms behind such enhancements are not yet fully understood. To further explore the role of nanoparticles on the enhanced mechanical properties of hydrogel nanocomposites, we used chemically crosslinked polyacrylamide hydrogels incorporating silica nanoparticles as the model system. Rheological measurements indicate that nanoparticle-mediated increases in hydrogel elastic modulus can exceed the maximum modulus that can be obtained through purely chemical crosslinking. Moreover, the data reveal that nanoparticle, monomer, and chemical crosslinker concentrations can all play an important role on the nanoparticle mediated-enhancements in mechanical properties. These results also demonstrate a strong role for pseudo crosslinking facilitated by polymer–particle interactions on the observed enhancements in elastic moduli. Taken together, our work delves into the role of nanoparticles on enhancing hydrogel properties, which is vital to the development of hydrogel nanocomposites with a wide range of specific mechanical properties.

## 1. Introduction

Hydrogels have recently emerged as potential candidates for various biomedical and biotechnological applications owing to their unique physical and biochemical properties. Composed of highly porous and hydrated networks, they allow cell encapsulation for tissue engineering applications and support the loading and release of various bioactive molecules for drug delivery applications [1,2,3]. The use of hydrogels in bioseparations and biosensing and as tissue-adhesives has also been recently proposed [4,5]. Latest advances in polymer chemistry and synthesis as well as progress in the development of interpenetrating polymer network hydrogels have opened up new possibilities in developing hydrogel-based biomaterials with advanced properties. However, their poor mechanical properties have been a significant barrier to their widespread adoption for these applications. Over three decades of research have shown that the addition of nanoscopic filler particles to a variety of polymer systems (melts, elastomers, hydrogels, etc.) can have a large effect on their mechanical properties [6,7,8,9]. Both experimental studies [10,11,12] and modeling analyses [13,14,15] have indicated that the enhancements in nanocomposite properties relative to those of pure polymers are due in large part to an increase in polymer crosslink/entanglement density mediated by strong interactions with nanoparticles.

In this study, we investigate whether nanoparticle-mediated enhancements in polymer mechanical properties extend to hydrogel nanocomposites. Current research investigating the effects of incorporating nanoparticles reports enhanced mechanical properties for hydrogel nanocomposites relative to neat hydrogels [16,17,18,19]. However, barring a few studies [16,20], little research has been performed to understand the role of chemical or covalent crosslinking on nanoparticle-mediated changes in hydrogel properties. Such studies are crucial to developing a quantitative understanding of how pseudo-crosslinking or crosslinking density plays a role in the reinforcements observed due to the addition of nanoparticles. In our study, we performed rotational rheological measurements to evaluate the changes in the elastic modulus of chemically crosslinked polyacrylamide (pAAm) hydrogels due to the addition of silica nanoparticles. pAAm hydrogels are synthesized as a networked structure of repeating acrylamide (AAm) subunits chemically crosslinked using the bifunctional crosslinking agent *N*,*N*′-methylenebisacrylamide (Bis). Silica nanoparticles (SiNPs) are known to hydrogen bond with polyacrylamide [21,22] and so we expect there to be a strong interfacial binding energy between the polymer and nanoparticle surface. Strong interactions between nanoparticles and polymer chains have been shown to facilitate nanoparticle-mediated reinforcement of hydrogels in previous studies [23]. Therefore, this system allowed us to evaluate the effects of nanoparticle-mediated physical crosslinking in comparison to chemical crosslinking, by varying the degree of both chemical- and nanoparticle-mediated crosslinking as well as the monomer concentration.

## 2. Results

### 2.1. Influence of Crosslinker Concentration on Hydrogel Elastic Modulus

Our initial experiments focused on the impact of crosslinker concentration on hydrogel modulus. pAAm hydrogels prepared using various monomer and crosslinker (AAm/Bis) ratios, as shown in Table 1, were characterized using rotational rheometry. When elastic modulus is plotted against the concentration of Bis, the hydrogel elastic modulus initially increases with crosslinker concentration for all concentrations of the monomer. However, above a threshold crosslinker concentration, the elastic modulus for each hydrogel reaches a plateau value (Table 1). 

Next, in Table 2, we show elastic modulus as a function of %*C_Bis_*, relative concentration of the crosslinker Bis, which is defined by Equation (1):

(1) $$\%C_{Bis}=\;\frac{m_{Bis}}{m_{Bis}+\;m_{AAm}}$$

where *m_AAm_* is the concentration of the monomer and *m_Bis_* is the concentration of the crosslinker.

For each hydrogel, irrespective of the monomer concentration, the threshold point occurs at the same relative crosslinker concentration (%*C_Bis_* = 4.76) (Supplementary Figure S1). Such saturation of hydrogel elastic modulus at a threshold relative chemical crosslinker concentration has been demonstrated previously [24], and has been shown to be the result of the formation of highly crosslinked “microgels” connected by a percolating network of linear polymer chains. Each microgel forms a mesoscale crosslink point, so that the overall crosslink density becomes a function of the number of microgels rather than the number of chemical crosslinking molecules. Since the kinetics of microgel formation are governed by the local concentration of the crosslinker, the number of microgels per unit volume reaches a maximum at a particular crosslinker concentration relative to the monomer [24,25].

### 2.2. Influence of Nanoparticles on the Elastic Modulus of Chemically Crosslinked Hydrogels

**Figure 1:**
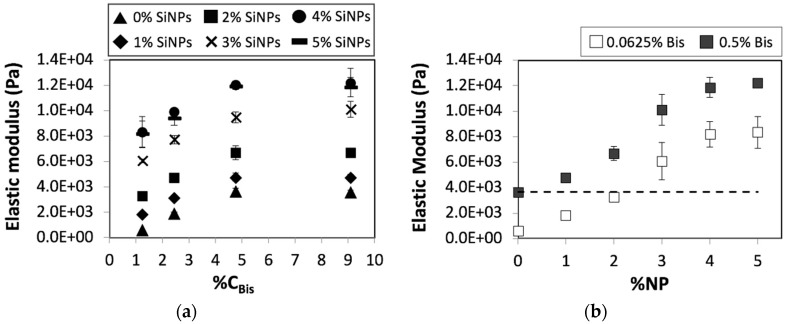
Elastic moduli of 5% pAAm hydrogels as a function of (**a**) relative crosslinker concentration (%*C_Bis_*) prepared using different concentrations of 4 nm silica nanoparticles—0% SiNPs (triangles), 1% SiNPs (diamonds), 2% SiNPs (squares), 3% SiNPs (Xs), 4% SiNPs (dashes), and 5% SiNPs (circles) and (**b**) nanoparticle concentration (%NP) prepared using different concentrations of the chemical crosslinker—0.0625% Bis (open squares) and 0.5% Bis (closed squares). Data shown are the mean of triplicate measurements ± standard deviation and have been repeated at least three times with similar results. Dashed line in panel (**b**) represents elastic modulus of 5% pAAm hydrogel prepared using 0.5% Bis and 0% SiNPs and acts as a guide to the eye.

We proceeded to study the influence of nanoparticles on the chemically crosslinked pAAm hydrogels. When silica nanoparticles were added to the pAAm hydrogel, we observed a concentration-dependent increase in the elastic modulus (Figure 1a). Moreover, the experiments revealed that addition of nanoparticles increase the elastic modulus beyond the maximum modulus observed for the purely chemically crosslinked system (Figure 1b). It is interesting to note that even at sub-saturation values of the relative crosslinker concentration, incorporation of nanoparticles allows enhancements beyond the plateau modulus achieved using chemical crosslinking (Figure 1b). This behavior was observed for hydrogels over a range of monomer concentrations. These results are consistent with previous studies, including our investigations that have indicated hydrogen bonding mediated interactions between pAAm chains and SiNP surfaces enable silica nanoparticles to serve as pseudo crosslinkers and increase the extent of crosslinking in the hydrogel network, thereby facilitating reinforcements in the mechanical properties [22,26].

**Figure 2:**
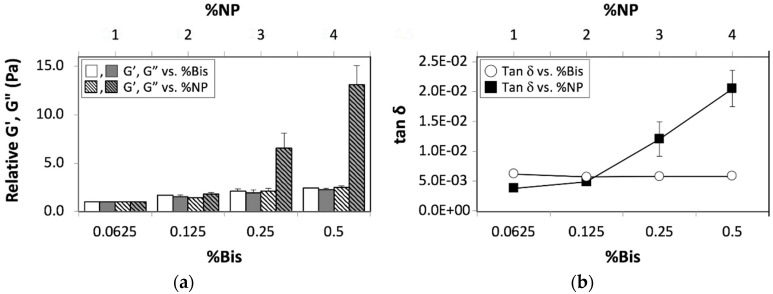
(**a**) Relative elastic (white bars) and viscous (grey bars) moduli of 5% pAAm hydrogels as a function of crosslinker concentration (%Bis) (solid bars) prepared using 1% 4 nm SiNPs and nanoparticle concentration (%NP) prepared using 0.0625% Bis (hashed bars), and (**b**) tan δ (ratio of G″/G′) as a function of the crosslinker (white circles) and nanoparticle concentration (black squares). Data shown are the mean of triplicate measurements ± standard deviation and have been repeated at least three times with similar results.

We also compared the effects of chemical crosslinking and nanoparticles on the viscous modulus, G″. These experiments revealed similar increases in the values of G′ and G″, when plotted against the concentration of Bis. However, we observed significant increases for G″ relative to G′, when plotted against the nanoparticle concentration (Figure 2a). Not surprisingly, the ratio of G″/G′ (tan δ) exhibited a significantly higher slope for tan δ plotted against the nanoparticle concentration compared to tan δ plotted against the concentration of Bis (Figure 2b). These experiments, therefore, indicate that nanoparticle-mediated reinforcements are independent from those mediated by chemical crosslinking. 

### 2.3. Influence of Chemical Crosslinking on Nanoparticle Mediated Enhancements of Hydrogel Elastic Modulus

**Figure 3:**
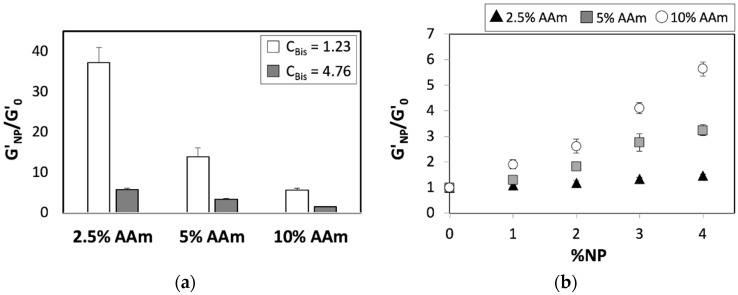
Relative elastic moduli of pAAm hydrogels as a function of (**a**) monomer concentration (%AAm) prepared using 5% 4 nm SiNPs and different concentrations of the chemical crosslinker—0.0625% Bis (white bars) and 0.5% Bis (grey bars) and (**b**) nanoparticle concentration for 2.5% pAAm (white circles), 5% pAAm (grey squares), and 10% pAAm (black triangles) hydrogels prepared using %*C_Bis_* = 4.76. Data shown are the mean of triplicate measurements ± standard deviation and have been repeated at least three times with similar results.

Finally, we explored the combined effects of chemical and nanoparticle-mediated crosslinking on the pAAm elastic modulus. We compared the enhancements afforded by the incorporation of nanoparticles (G′_NP_/G′_0_, the ratio of elastic modulus of hydrogels that incorporated or did not incorporate nanoparticles) over a range of both crosslinker and monomer concentrations. Interestingly, these experiments revealed a diminishing impact of nanoparticles on the elastic modulus at higher chemical crosslinker and monomer concentrations (Figure 3 and Supplementary Figure S2). The decreasing role of nanoparticles with increasing crosslinker concentrations indicates a saturation point in the overall hydrogel crosslinking density and thereby enhancements in mechanical properties afforded by either covalent- or pseudo crosslinking and a combination thereof.

To better understand the role of monomer concentration on the nanoparticle-mediated enhancements in elastic modulus, we defined a new variable (%*C_NP_*) that refers to the concentration of SiNPs relative to the monomer concentration in a manner similar to %*C_Bis_* (Equation (2)).

(2) $$\%C_{NP}=\;\frac{m_{NP}}{m_{NP}+\;m_{AAm}}$$

where *m_AAm_* is the concentration of the monomer and *m_NP_* is the concentration of the nanoparticles.

**Figure 4:**
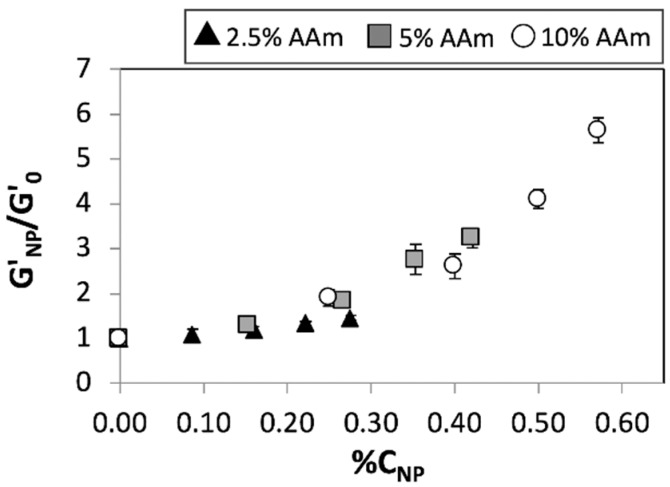
Relative elastic moduli of 2.5% pAAm (white circles), 5% pAAm (grey squares), and 10% pAAm (black triangles) hydrogels prepared using %*C_Bis_* = 4.76 as a function of relative nanoparticle concentration (%*C_NP_*). Data shown are the mean of triplicate measurements ± standard deviation and have been repeated at least three times with similar results.

The enhancements in elastic modulus afforded by the incorporation of nanoparticles for various AAm concentrations at saturation (%*C_Bis_* = 4.76) and sub-saturation (%*C_Bis_* = 1.23) concentrations of Bis, previously plotted against the absolute nanoparticle concentration (%NPs), were plotted against the relative nanoparticle concentration (%*C_NP_*) (Figure 4 and Supplementary Figure S3). The enhancements mediated by nanoparticles for various monomer concentrations (Figure 3b) can be collapsed onto a single curve (Figure 4) upon the introduction of %*C_NP_*, which accounts for relative reinforcement due to nanoparticles at specific concentrations of the chemical crosslinker. These results thereby indicate that the nanoparticle mediated enhancements in hydrogel modulus scales with the relative nanoparticle concentration, not unlike the enhancements mediated by chemical crosslinking.

### 2.4. Influence of Hydrophobic Side Chains on the Hydrogel Backbone on Nanoparticle Mediated Enhancements

**Figure 5:**
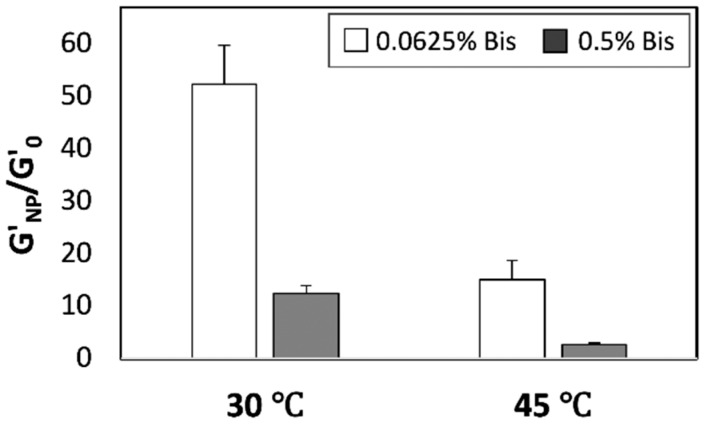
Relative elastic moduli of 5% pNIPAAm hydrogels prepared using 0.0625% Bis (white bars) or 0.5% Bis (grey bars), and 5% SiNPs at different temperatures. Data shown are the mean of triplicate measurements plus standard deviation and have been repeated at least three times with similar results.

To validate our hypothesis that suggests a saturation point in the overall hydrogel crosslinking density mediated by a combination of chemical (Bis) and physical (SiNPs) crosslinking, we repeated the mechanical characterization studies using poly(*N*-isopropylacrylamide) (pNIPAAm) hydrogels. pNIPAAm is a temperature-responsive hydrogel with a lower critical solution temperature (LCST) of ca. 32 °C in aqueous solutions [27,28]. The presence of hydrophobic side groups on the pNIPAAm hydrogel backbone leads to increased polymer–polymer interactions (and therefore increased physical crosslinking) at temperatures above LCST and thereby higher elastic modulus. Our initial experiments indicated that pNIPAAm hydrogels incorporating nanoparticles continue to exhibit temperature-dependent changes in mechanical properties, i.e., a reversible phase transition at temperatures greater than 32 °C. These experiments also indicated that pNIPAAm-SiNP hydrogels exhibit a higher elastic modulus compared to neat hydrogels (without nanoparticles). Interestingly, the nanoparticle-mediated enhancements in the elastic modulus were higher at temperatures <LCST relative to those at temperatures >LCST (Figure 5). This result is consistent with our hypothesis; if the reduced role of nanoparticles at higher crosslinker concentrations is a result of saturation in the overall hydrogel crosslinking density, then a similar effect (i.e., attenuated role of nanoparticles) should be observed in the presence of other sources of crosslinking as well. In the case of pNIPAAm hydrogels, an increase in physical crosslinking mediated by the hydrophobic side chains leads to saturation in overall crosslinking density and thereby an upper limit to enhancements achieved through the incorporation of nanoparticles. 

## 3. Discussion

This study sought to examine the contributions of nanoparticle-mediated physical crosslinking to the elastic modulus of chemically crosslinked hydrogels, using pAAm-SiNP composites as the model system. Results from the rheological measurements showed that hydrogel elastic modulus positively correlated with both Bis and SiNP concentration. These experiments also indicated that a combination of chemical and physical crosslinking led to enhancements in elastic modulus that were greater than with either alone, which is consistent with previous studies [16,26,29]. Additional experiments suggested that the nanoparticle-mediated enhancements behave differently to those mediated by chemical crosslinking. However, we observed an upper limit to the gains in elastic modulus achievable through a combination of Bis- and SiNP-mediated crosslinking, suggesting the existence of a saturation point for the combined crosslinking density (i.e., the sum of crosslinking densities achieved through either chemical and physical means). To confirm the existence of a ‘global’ saturation point, beyond the ‘local’ saturation behaviors observed for either Bis- or SiNP-mediated enhancements, we introduced an additional source of physical crosslinking—pAAm functionalized with hydrophobic side groups that lead to increased polymer–polymer interactions at elevated temperatures. These experiments revealed attenuated enhancements mediated by nanoparticles at temperatures >LCST, consistent with the hypothesis that saturation in the overall crosslinking density can lead to upper limits in enhancements in modulus achieved through the addition of nanoparticles. 

To better understand the observed role of nanoparticles on the hydrogel elastic modulus, we borrowed lessons learned from more classic polymer nanocomposite systems [6,7,8,10,11,12,13,14,15]. Certain traditional reinforcement models, such as simple filler effects, jamming theory, and fractal percolating networks, fail to explain the results from our studies. While we observe a linear relationship between elastic modulus and SiNP concentration for concentrations less than 3%, we observe saturation behavior at higher concentrations irrespective of monomer concentration. Furthermore, we have previously shown that the nanoparticle size also has an effect on the degree of enhancement, which means the enhancements cannot be explained by simple volume fraction arguments [30]. Jamming theory describes the response of a polymer to the drag friction caused by particles; however, it is unlikely for jamming to contribute significantly towards the elastic modulus of pAAm nanocomposites in our system given the dilute concentration of nanoparticles (0–5% SiNPs) [31,32]. The same can be said for fractal percolating structures that may form due to direct interactions among silica nanoparticles, as the likelihood that these arrangements will play a meaningful role at/below particle concentrations of 5% is low [33,34,35]. Other models that either explain reinforcement due to retardation of polymer dynamics in the interfacial zone around the nanoparticles, or the number of crosslinks or entanglements in a polymer network lend more support to our observations. In the cases where the polymer–particle interaction energy is much larger than the polymer–polymer interaction energy, the time scale of the movements of short lengths of polymer chains (the Rouse regime) is slowed down within a region extending from the particle surface to the limit of the polymer–particle energy well [13]. The result is an increase in elastic modulus that is proportional to the volume fraction of the interfacial zone and the magnitude of interfacial energy. Since volume fraction of the interfacial zone is positively correlated to particle concentration and negatively correlated to particle size, our results (i.e., increasing modulus with increasing SiNP concentration and decreasing SiNP size) can be explained by, albeit qualitatively, using the retardation polymer dynamics in the interfacial zone model. From a polymer–particle interaction standpoint, Flory’s rubber elasticity theory may also provide a possible mechanism. Flory’s network theory states that elastic modulus is proportional to the density of effective junctions [24,36]. As indicated in previous studies, non-covalent interactions between silica nanoparticles and polyacrylamide can serve to increase the number of effective crosslinks in the network and thereby lead to increases in elastic modulus, consistent with Flory’s theory.

In addition to providing further insight into how nanoparticles mediate reinforcements in hydrogel elastic modulus, this study also contributes to the development of applications that may directly benefit from these improvements; examples include tissue engineering, drug delivery, wound dressings, and biosensing [17,37]. Some of these applications, especially those related to sensing, may also benefit from enhancements in the chemical and biological properties of the hydrogel. Nanoparticles may be functionalized with biomolecules prior to their incorporation into the hydrogel network to endow the hydrogels with specific biochemical characteristics in addition to improving their mechanical strength. Furthermore, new applications may also be realized by introducing and/or tailoring additional hydrogel properties such as thermal, electrical, optical, and magnetic characteristics. Several studies have already demonstrated the use of nanoparticles to develop polymer composites with conductive properties for battery cathodes and microelectronics [38], with light responsive properties for therapy [39], and with magnetic properties for electromagnetic interference shielding [40]. As we continue to further explore the use of nanoparticles to improve hydrogel properties, we can expect that the applications realized for traditional polymer composites may also translate to nanocomposites prepared using hydrogels.

## 4. Materials and Methods 

### 4.1. Materials

All the materials for the polymerization reaction, acrylamide (AAm, monomer), initiator, ammonium persulfate (APS, initiator), *N*,*N*,*N*′,*N*′-tetramethylethylenediamine (TEMED, catalyst), and *N*,*N*′-methylenebis(acrylamide) (Bis, crosslinker), as well as *N*-isopropylacrylamide (NIPAAm, monomer for preparing thermoresponsive hydrogels) were purchased from Sigma Aldrich (St. Louis, MO, USA) and used as received. Tris-HCl buffer (pH 7.2) was obtained from Life Technologies (Carlsbad, CA, USA) and binzil silica nanoparticle colloid solution with mean particle size of 4 nm was obtained as a gift from AkzoNobel Pulp and Performance Chemicals Inc. (Marietta, GA, USA). 

### 4.2. Polymerization Reaction

Chemically crosslinked pAAm or pNIPAAm hydrogels were prepared as previously reported [26,41]. Briefly, the monomer (AAm or NIPAAm) and crosslinker (Bis) stocks were diluted to their desired concentrations in pH 7.2, 250 mM Tris-HCl buffer, followed by the addition of TEMED (0.1% of the final reaction volume) and 10% *w*/*v* APS solution (1% of the final reaction volume). For nanocomposite hydrogels, various amounts of silica nanoparticles (SiNPs) were added to the reaction mixture prior to the addition of APS and TEMED. Due to solubility limits, the maximum nanoparticle concentration used was 5% *w*/*v*. Polymerization reactions were performed at 25 °C between parallel plates of the rheometer cell to minimize exposure to air as oxygen inhibits the free radical polymerization reaction. 

### 4.3. Measurement of Hydrogel Elastic Modulus

Rheological measurements of the hydrogels were carried out, as previously described, using the MCR302 rotational rheometer (Anton Paar, Graz, Austria) [26,41]. Briefly, 500 μL of a well-mixed reaction mixture was pipetted onto the lower plate of the rheometer and the upper plate was lowered until the desired gap distance (1 mm) was achieved. Amplitude sweeps at a constant frequency of 1 Hz were then carried out to ensure measurements were carried out in the linear viscoelastic regime of the hydrogels. Next, dynamic sweep tests over frequencies ranging from 0.1–100 Hz were recorded in the linear viscoelastic regimes (strain amplitude = 0.01) to determine the shear storage modulus. Final hydrogel parameters were determined by following the gelation for 90 min at 1 Hz and 1% strain for all samples. For the temperature studies using pNIPAAm hydrogels, the elastic modulus was measured at 30 °C and 45 °C using the conditions described above. Relative elastic and viscous moduli were calculated by normalizing the values for pAAm-SiNP hydrogels (G′_NP_ and G″_NP_) to the corresponding values for control pAAm gels (G′_0_ and G″_0_). 

## Figures and Tables

**Table 1:** Elastic modulus, G′, for neat hydrogels for various monomer and crosslinker ratios.

%Bis	Elastic Modulus, G′ ^a^		
	10% AAm	5% AAm	2.5% AAm
1	(1.96 ± 0.14) × 10^4^	(3.72 ± 0.13) × 10^3^	(4.22 ± 0.42) × 10^2^
0.5	(1.98 ± 0.36) × 10^4^	(3.57 ± 0.19) × 10^3^	(4.07 ± 0.49) × 10^2^
0.25	(1.03 ± 0.03) × 10^4^	(3.67 ± 0.27) × 10^3^	(4.27 ± 0.22) × 10^2^
0.125	(5.78 ± 0.86) × 10^3^	(1.95 ± 0.09) × 10^3^	(4.14 ± 0.59) × 10^2^
0.0625	(2.67 ± 0.35) × 10^3^	(5.98 ± 0.17) × 10^2^	(4.78 ± 0.81) × 10^1^

^a^ Each data point represents an average of triplicate measurements.

**Table 2:** Elastic modulus, G′, for neat hydrogels for various monomer and relative crosslinker ratios.

%*C_Bis_*	Elastic Modulus, G′ ^a^		
	10% AAm	5% AAm	2.5% AAm
9.09	(1.96 ± 0.14) × 10^4^	(3.57 ± 0.19) × 10^3^	(4.27 ± 0.22) × 10^2^
4.76	(1.98 ± 0.36) × 10^4^	(3.67 ± 0.27) × 10^3^	(4.14 ± 0.59) × 10^2^
2.44	(1.03 ± 0.03) × 10^4^	(1.95 ± 0.09) × 10^3^	(4.78 ± 0.81) × 10^1^
1.23	(5.78 ± 0.86) × 10^3^	(5.98 ± 0.17) × 10^2^	N/A ^b^

^a^ Each data point represents an average of triplicate measurements. ^b^ This condition was below the limits of reliable detection using rotational rheology.

## Supplementary Materials

The following are available online http://www.mdpi.com/2079-4991/8/11/882/s1. Figure S1: Elastic modulus for neat hydrogels for various monomer and relative crosslinker ratios. Data shown are the mean of triplicate measurements ± standard deviation and have been repeated at least three times with similar results; Figure S2: Relative elastic moduli of pAAm hydrogels as a function of nanoparticle concentration for 2.5% pAAm (white circles), 5% pAAm (grey squares), and 10% pAAm (black triangles) hydrogels prepared using %C_Bis_ = 1.23. Data shown are the mean of triplicate measurements ± standard deviation and have been repeated at least three times with similar results; Figure S3: Relative elastic moduli of 2.5% pAAm (white circles), 5% pAAm (grey squares), and 10% pAAm (black triangles) hydrogels prepared using %C_Bis_ = 1.23 as a function of relative nanoparticle concentration (%CNP). Data shown are the mean of triplicate measurements ± standard deviation and have been repeated at least three times with similar results.
