# Supplementary material for: Exploring the Role of Nanoparticles in Enhancing Mechanical Properties of Hydrogel Nanocomposites
Source: Nanomaterials (Basel). 2018 Oct 29;8(11):882. doi: 10.3390/nano8110882 (PMC6265757; doi:10.3390/nano8110882)
Supplement: Supplementary file 1 [file nanomaterials-08-00882-s001.pdf]

nanomaterials

Supplementary Information

Understanding the role of nanoparticles in enhancing mechanical properties of hydrogel nanocomposites

Josergio Zaragoza[1], Scott Fukuoka[1], Marcus Kraus[1], James Thomin[2] and Prashanth Asuri[1,*]

[1] Department of Bioengineering, Santa Clara University, Santa Clara, CA 95053, USA; j1zaragoza@scu.edu; sfukuoka@scu.edu; mkraus1@scu.edu

[2] Department of General Sciences, Northwest Florida State College, Niceville, FL 32578, USA; thominj@nwfsc.edu

* Correspondence: asurip@scu.edu; Tel.: +1-408-551-3005

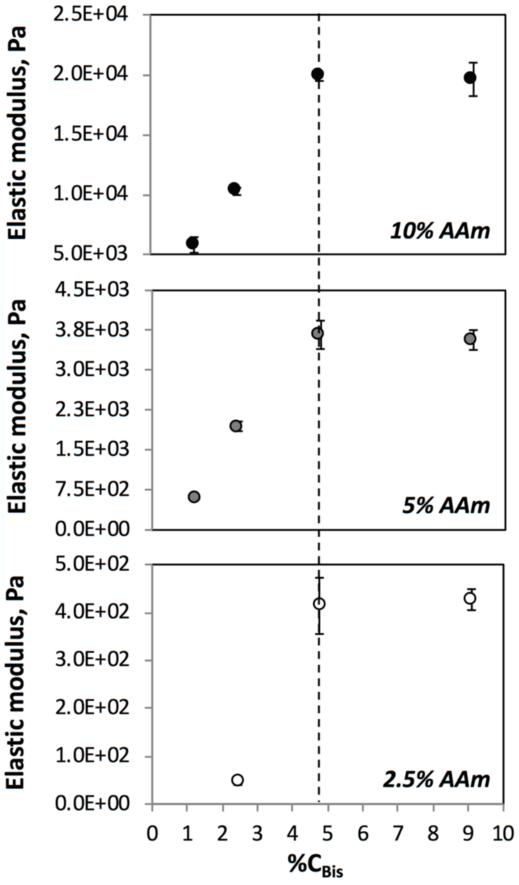

Figure S1. Elastic modulus for neat hydrogels for various monomer and relative crosslinker ratios. Data shown are the mean of triplicate measurements ± standard deviation and have been repeated at least three times with similar results.

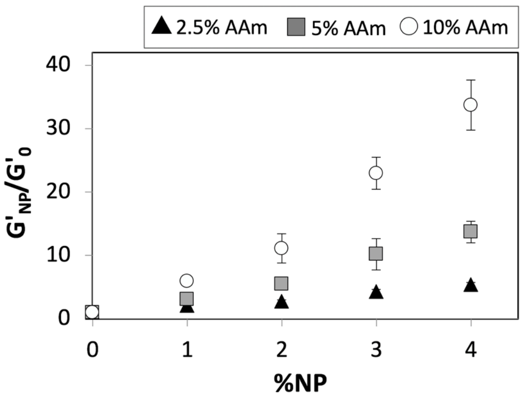

Figure S2. Relative elastic moduli of pAAm hydrogels as a function of nanoparticle concentration for 2.5% pAAm (white circles), 5% pAAm (grey squares), and 10% pAAm (black triangles) hydrogels prepared using $\%C_{Bis}$ = 1.23. Data shown are the mean of triplicate measurements ± standard deviation and have been repeated at least three times with similar results.

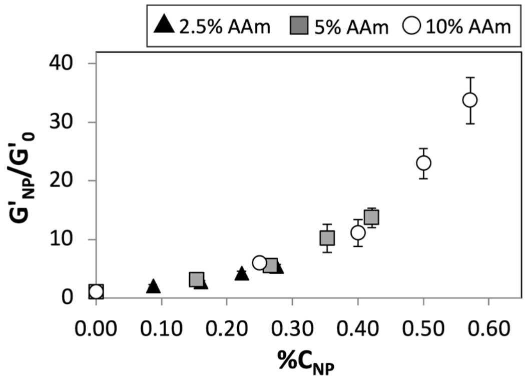

Figure S3. Relative elastic moduli of 2.5% pAAm (white circles), 5% pAAm (grey squares), and 10% pAAm (black triangles) hydrogels prepared using $\%C_{Bis}$ = 1.23 as a function of relative nanoparticle concentration ($\%C_{NP}$). Data shown are the mean of triplicate measurements ± standard deviation and have been repeated at least three times with similar results.
